# The Book World of Medicine and Science

**Published:** 1897-07-31

**Authors:** 


### The Book World of Medicine and Science.

Retinoscopy. By James Thorington, M.D , Adjunct Professor of Diseases of the Eye in the Philadelphia Polyclinic, &c. (Philadelphia: Blakiston. London: Kegan Paul. 1897. Pp. 66, with 24 illustrations. 1 dol.)

This is a wholly valuable little manual on a subject of great and increasing importance, for we have no hesitation in agreeing with the author that properly-conducted retinoscopy is by far superior to the subjective method of estimating errors of refraction. But to give accurate results, the investigation must be conducted in a systematic and, above all, in a uniform manner, and for this purpose no better guide than Dr. Thorington's book could be desired. He adopts, and with good reason, the use of the plane mirror, employing one 2cm. in diameter on a 4cm. blackened metal disc and with a sight hole of 2mm. diameter. This is held as near as possible to the source of light and at a measured distance of one metre from the observed eye. The light is obtained from an Argand burner surrounded by an asbestos screen devised by the author, and having apertures of various diameters in it; the most suitable aperture is found to be one of ½ to 1cm. Working in this exact manner, Dr. Thorington is able to give a complete physical demonstration of the results obtained. As the distance between the retinoscope and the observed eye is one metre, a sphere of one diopter must always be added to the neutralising lens obtained by investigation in the dark room. The emmetropic or infinity correction is thus infallibly obtained and can be checked, if desired, by the subjective test. The author invariably uses a mydriatic, which is also essential in the reduction of retinoscopy to an exact science. This is, indeed, his object, and he has described the method most satisfactorily, in the clearest possible language and with the aid of most simple and instructive diagrams. He is temperate in his recommendation of the method, but convincing in his proof of its superiority to all others. We can unhesitatingly recommend the book to all who wish to acquire or to perfect themselves in the art of which it treats; to the country practitioner, far removed from any school and plagued by the ignorance and stupidity of many of his patients, it will be a real boon, enabling him to teach himself to be independent of the fallacies and annoyances which are always bound to accompany the subjective mode of correcting errors of refraction.

The Surgical Diseases of Children. By Edmund Owen, M.B., F.R.C.S., Senior Surgeon to the Hospital for Sick Children, Great Ormond Street; Senior Surgeon to and Lecturer on Surgery at St. Mary's Hospital. Third edition. Revised and enlarged. (London: Cassell and Co. 1897. Price 21s.)

We have read this book with very great pleasure, and can strongly recommend it to those who wish for a reliable guide to the subject of the surgery of infancy and childhood. The book has been considerably enlarged, and its contents have been carefully revised so as to bring them up to date. In the introductory chapter many wise bits of advice are given, the outcome of personal dealings with the little folk to whose ailments the author has addressed himself. Among other points we would cordially agree with the statement that the early morning is the best time for operating on children, as they are thus less disturbed by being kept without food. The following suggestion is peculiarly neat, although rather hard upon the "other man." After saying that the anæsthetic should be administered before the child is taken out of bed, the author proceeds: "In private practice I keep out of sight until the child is insensible and allow the anæsthetist to bear the charge of having 'hurt' him; in the matter of subsequent dressings I come as a sympathetic friend to heal the wound which 'the other man' has inflicted." A much-needed caution is given as to the extreme susceptibility which some children show to the action of the various substances used as antiseptics. "Not long ago," he says, "I saw in consultation a delicate child who had been seriously affected by the application of, as I was informed, the ordinary wet dressings of carbolic lotion previous to the performance of an operation; extensive gangrene followed the application." He especially draws attention to the occasional occurrence of iodoform poisoning where that drug is used.

The question when to perform tracheotomy is one which causes many surgeons much anxiety, which may perhaps be somewhat relieved by the assurance that, when there are signs of obstruction in diphtheria, if there be a doubt as to whether the operation may be still further delayed it will be better to perform it at once, and by the further dictum that "operation will not unfavourably prejudice the child's chances—that is quite certain."

At first sight one might think that the very large and important subject of tuberculosis has a somewhat meagre amount of space allotted to it, squeezed in as it is between hæmophilia and rickets; but as the reader passes on he finds that the subject crops up again and again under other headings, such as caries and joint diseases. We notice a curious omission in the section dealing with adenoids. Many details are given as to the arrangements for the operation, the position of the child, and such like matters, but as to the operation itself there is only the following incidental reference: "Whilst the vegetations are being scraped away with the nail of the left forefinger, and with a slender Meyer's ring-knife which is passed along the nasal floor, the head is steadied and supported by the assistant." As beyond this there is no special description of the operation, perhaps it would be unfair to cavil at the absence of all reference to methods of operating adopted by other surgeons. Still, we can hardly admit that the mention of the ring-knife which is passed along the nasal floor is a fair presentment of the instrumental aid available for the removal of these growths. The chapter on hip-joint disease is good, and is illustrated by some very convincing diagrams, showing the relation between the apparent shortening or lengthening of the limb, which is so often met with, and the real condition of affairs. Mr. Owen urges the importance of performing puncture of the capsule of the joint in cases where there is effusion hindering the limb from being placed in a proper position. The puncture may be made either through the gluteus maximus or by working inwards from below the anterior superior spine of the ilium.

It must not be imagined that this work is intended to take the place of larger works on surgery, or that it gives a complete description of every surgical malady which may occur in childhood, but so far as it deals with the peculiarities which mark these diseases when they do so occur and the points in which their treatment is influenced by the youth of the patients, it is an admirable treatise. Both the printing and the illustrations are very good, and among the latter we would especially draw attention to the chromo-lithograph of a case of tuberculous dactylitis, and to one representing a pitiable infant suffering from extreme rickets. No doubt it is extreme, but it is true to life.

