# Books Received

**Published:** 1897-08-07

**Authors:** 


### A CORRECTION.

In our notice of "Books Received" last week, the following should have been inserted: J. and A. Churchill, "Convergent Strabismus and its Treatment," by Edwin Holthouse.

